# A tailored bioactive 3D porous poly(lactic-acid)-exosome scaffold with osteo-immunomodulatory and osteogenic differentiation properties

**DOI:** 10.1186/s13036-022-00301-z

**Published:** 2022-08-22

**Authors:** Yi Zhang, Mengjie Huo, Yi Wang, Lan Xiao, Jianmei Wu, Yaping Ma, Dingmei Zhang, Xuemei Lang, Xin Wang

**Affiliations:** 1 Department of Hygiene Toxicology, Zunyi Medical University, Zunyi, 563000 Guizhou China; 2 Department of Orthopaedic Surgery, Affiliated Hospital of Zunyi Medical University, Zunyi, 563003 Guizhou China; 3 School of Mechanical, Medical & Process Engineering, Centre for Biomedical Technologies, Queensland University of Technology (QUT), Brisbane, QLD 4000 Australia; 4 Australia China Centre for Tissue Engineering and Regenerative Medicine, Kelvin Grove, Brisbane, Queensland 4059 Australia; 5 Department of Pre-hospital Emergency, Central Hospital of Chongqing University / Chongqing Emergency Medical Center, Chongqing, Chongqing, 400014 China

**Keywords:** MSC-Exo, Bioactive 3D PLA scaffold, Macrophages, Immunoregulation, Osteogenesis

## Abstract

Polylactic acid (PLA) is a versatile and biodegradable scaffold widely used in biomedical fields to repair tissue defects. Exosomes derived from mesenchymal stem cells (MSCs) are nano-sized extracellular vesicles, which play an important role in tissue engineering in recent years. The primary focus of this study was to develop a bioactive 3D PLA scaffold using exosome-based strategy to improve its osteogenic and immunoregulatory potential. We firstly successfully isolated MSC-derived exosomes (MSC-Exo). Morphological analysis revealed that MSC-Exo exhibits a typical cup-shaped morphology with high expression of exosomal marker CD63. MSC-Exo internalization into recipient cells were also investigated using flow cytometry and confocal laser scanning microscopy. Porous 3D PLA scaffold coated MSC-Exo were used for immunoregulatory and osteogenic testing. Exosomes released from 3D PLA scaffold were validated in RAW264.7 and hBMSCs. The cell proliferation and live/dead assay indicated high biocompatibility for PLA-Exo scaffold. Additionally, PLA-Exo scaffold could reduce the pro-inflammatory marker expression and reactive oxygen species (ROS) production, indicating potential immunoregulatory potential. It is also confirmed that PLA-Exo scaffold could potentiate osteogenic differentiation in the osteogenesis assay. In conclusion, our results demonstrate this bioactive 3D-printed PLA scaffolds with MSC-Exo modification holds immunoregulatory potential and favor osteogenic differentiation, thus having potential applications in bone tissue regeneration.

## Introduction

Large bone defects or non-union bone fractures caused by musculoskeletal tumors, traumatic injury, or under various pathological conditions, represent a fundamental challenge for orthopedic surgeons [1]. To date, various reconstructive options, including autologous iliac grafting, allogeneic bone graft, induced membrane technique, as well as using various bioactive materials, have gained acceptance in orthopedic practice to treat large bone defects [2]. Autografts containing native tissues and a vascularization bed remain the gold standard technique to reconstruct large bone defect due to its inherent osteoinductive and biocompatible properties [3, 4]. However, the main disadvantages are labor-intensive surgery, limited sources, and possible donor site complications [4]. The incident of non-union bone fractures will continue to rise with the aging populations, thus causing huge social and economic burdens.

Exosomes (Exo), with an average size of 40-160 nm, are a type of extracellular vesicles secreted by most eukaryotic cells [5]. Since its discovery in 1980s, exosomes have attracted increasing attentions in recent years to contribute intercellular communication under various physiological and pathological conditions [6]. Surrounded by a lipid bilayer membrane, exosomes are functional vehicles that carry a bioactive cargo of proteins, nucleic acids (DNA, mRNA, microRNA, circular RNA), lipids, and metabolites, etc., which play an important role in reprogramming recipient cell function and behavior [7]. In recent years, therapeutic intervention mediated by exosomes with immunomodulatory properties have attracted increasing attentions to accelerate skeletal tissue repair. Mesenchymal stem cells (MSCs) are nonhematopoietic fibroblast-like multipotent adult stem cells derived from adult tissues with self-renewal potential [8]. Over the past decades, the application of exogenous bone marrow derived MSCs, along with other bioactive molecules, and composite scaffolds, have exhibited enormous potential for regenerating skeletal tissue [9, 10]. Additionally, therapeutic intervention mediated by exosomes from MSCs, are being explored as novel cell-free alternative to cellular therapy in regenerative medicine [11]. Administration of MSCs derived exosomes has been reported to be efficacious in promoting skeletal muscle regeneration [12], cartilage repair [13], and bone fracture healing [14], and cutaneous wound healing [15]. Additionally, the broad-spectrum therapeutic efficacy of MSC-derived exosomes have also been validated in multiple disease models, including carbon tetrachloride (CCl4)-induced liver injury [16], myocardial ischemia/reperfusion injury [17], LPS-induced acute respiratory distress syndrome [18], and graft-versus-host disease [19].

Numerous biocompatible and biodegradable polymeric materials, such as polycaprolactone (PCL), poly(acrylonitrile butadiene styrene) (ABS), polylactic acid (PLA), and polyglycolic acid (PGA), etc. are widely used in the field of regenerative medicine [20]. PLA has been increasingly utilized to construct 3D-printed bone implants [21]. Despite its mechanical stability, and cyto-compatibility advantage, further surface modification is still needed to increase its bioactivity. In this study, we developed an exosome-based surface modification strategy to modify the PLA surface to improve it osteogenic and immunoregulatory potential. Our results indicate that PLA/MSC-Exo scaffold may serve as a potential therapeutic scaffold for hard tissue regeneration.

## Materials and methods

### Cell culture and exosome isolation

To obtain exosomes, the human bone marrow-derived mesenchymal stromal cells (hBMSCs, ATCC® PCS-500-012™) were used in this study. Cells were cultured in Dulbecco’s Modified Eagle’s Medium (DMEM; Life Technologies Pty Ltd., China) supplemented with 10% fetal bovine serum (FBS; Biological Industries, LTD, Beit Haemek, Israel), and 1% (v/v) penicillin/streptomycin (Solarbio, Beijing, China) in atmosphere of 5% CO2 at 37 °C. For conditioned medium (CM) collection, hBMSCs (passage 3) were seeded in T75 flasks and were cultured to 90% confluence at 37 °C. After rinsing thrice with phosphate-buffered saline (PBS), cells were cultured with 10 mL DMEM supplemented with 10% exosome-depleted FBS at 37 °C for 24 h in order to collect CM [22]. The collected CM was pooled together before exosomes isolation. Exosomes were isolated according to published guidelines [23]. First, CM was filtered through 0.22 μm filters to remove live cells and other large membranous structures. Second, CM was centrifuged at 300×*g* at 4 °C for 10 min to pellet any remaining live cells. Third, CM was transferred to new tubes and centrifuged at 2,000×*g* at 4 °C for 20 min. Then the CM was centrifuged in a 45Ti rotor (Beckman) at 10,000×*g* at 4 °C for 40 min. The supernatant was spun at 100,000×g at 4 °C for 90 min to pellet exosomes. The exosome pellets were resuspended in PBS, aliquoted and stored at − 80 °C immediately until further analysis.

### Transmission electron microscopy (TEM)

Exosomes (5 μL) were mounted on TEM copper grids (200 mesh and coated by formvar carbon film) for 5 min at room temperature and stained with 1% uranyl acetate for 20 s. Excess uranyl acetate was removed by rinsing with deionized water and samples were dried using Whatman filter paper before imaging. A JEM-1400, JOEL TEM was used to image exosome samples at a voltage of 80 kV.

### Western blot

To identify the exosome marker, samples were resuspended in RIPA lysis buffer and analyzed by Western blot. Proteins were separated using 10% sodium dodecyl sulfate-polyacrylamide gel electrophoresis (SDS-PAGE) and transferred to a polyvinylidene difluoride (PVDF) membrane. The membrane was blocked with Odyssey® Blocking Buffer (LI-COR Biotechnology, USA) for 1 h. Membranes were incubated with the following primary antibody: Anti-CD63 Antibody (E-12) (1:100, sc-365,604, Santa Cruz). The following secondary antibody was used: IRDye® 680RD goat anti-mouse IgG (H + L) (1:10,000; LI-COR Biotechnology, USA). The protein signal intensity was detected using Odyssey infrared Imaging System (LI-COR Biotechnology, USA).

### Exosome labeling and cellular uptake of exosomes

Exosome labeling was performed using PKH26 red fluorescent cell linker kit for general cell membrane labeling according to the manufacturer’s instruction (PKH26GL-1KT, Sigma, China). PKH26-labeled exosomes were incubated with recipient cells for 24 h at 37 °C. After treatment, cells were washed with PBS twice, trypsinized using 0.25% trypsin-EDTA, and analyzed using a BD Caliber flow cytometer. The mean fluorescence intensity (MFI) was calculated according to the flow cytometry data.

For fluorescence imaging, cells were washed with PBS, fixed with 4% paraformaldehyde, permeabilized using 0.25% Triton, and stained with Alexa Fluor 488-labeled phalloidin in dark for 1 h followed by DAPI staining. Images were captured using a confocal laser scanning microscope with a × 40 objective (Leica DM IRB; Leica, Wetzlar, Germany).

### Preparation of porous PLA and MSC-Exo scaffolds

The patterns for 3D printed scaffolds were designed using AutoCAD software (Autodesk, Inc., San Rafael, CA, USA) and saved as stereolitography (.stl) file. PLA filament (diameter 1.75 mm) was used to fabricate layer-by-layer 3D scaffolds with a customized 3D printer as described previously [21]. Briefly, PLA filament (1.75 mm diameter) was fed directly into the printer head and extruded via the printing nozzle at 200 °C. The scaffolds were printed with a 10 mm diameter, 4 mm height with a pore diameter of ~ 500 μm. Poly(dopamine)-based surface modification technique was used to firstly modify the PLA scaffold based on our previous studies [22, 24]. Briefly, poly(dopamine) (PDA) coating was achieved by using 4 mg/ml dopamine hydrochloride in 10 mM pH =8.5 Tris buffer for 1 h with stirring before rinsing with Milli-Q water. For exosome coating, exosomes (10 μg in terms of protein) were incubated with PLA scaffold for 1 h at room temperature.

### Exosome internalization study

Exosome internalization study was performed according to a previous study [25]. In brief, PKH26 red fluorescent cell linker kit for general cell membrane labeling was used to label exosomes onto PLA scaffold. Cells were seeded on 24-well tissue culture-treated coverslip overnight, then co-cultured with different scaffolds for different time points (6 h and 24 h) at 37 °C. Cells were washed with PBS, fixed with 4% paraformaldehyde, permeabilized using 0.25% Triton, and stained with Alexa Fluor 488-labeled phalloidin in dark for 1 h followed by DAPI staining. Images were captured using a confocal laser scanning microscope with a × 40 objective (Leica DM IRB; Leica, Wetzlar, Germany).

### Macrophage proliferation and live/dead assay

MTT cell proliferation assay was used as a metabolic activity indicator for cell viability. Briefly, Raw264.7 cells were seeded on 24-well tissue culture-treated cover slips overnight, then rinsed with PBS, then co-cultured with different scaffolds. On day 1 and day 3, 5 mg/ml 3-(4, 5-dimethylthiazol-2-yl)-2, 5-diphenyl tetrazolium bromide (MTT, Sigma, China) was added to the wells and incubated for additional 4 h at 37 °C. One hundred microliter dimethyl sulfoxide (DMSO) was used to dissolve formazan generated during the incubation. The absorbance of the sample was measured using a microplate reader at 570 nm.

Live/dead staining was performed using staining solution containing 5 mg/mL FDA (fluorescein diacetate, green) and 2 mg/mL PI (propidium iodide, red) according to our previous study [22]. On day 1 and day 3, cells were incubated with the staining solution at room temperature. After washing with PBS, the samples were viewed using an inverted fluorescence microscope with a × 10 objective (Leica, Wetzlar, Germany).

### Macrophage polarization

Macrophages were activated with LPS as described previously [22]. Briefly, macrophages were seeded on 24-well tissue culture coverslip overnight, then stimulated with 1000 ng/ml of Lipopolysaccharide (LPS, *Escherichia coli* 0111: B4, Sigma, China) for 12 h. Cells were rinsed with PBS trice, then co-cultured with different scaffolds. After 24 h incubation, cells were washed with PBS, fixed with 4% paraformaldehyde, and stained with Alexa Fluor 594-labeled phalloidin for 1 h followed by DAPI staining. Images were acquired using a confocal laser scanning microscope with a × 40 oil objective (Leica DM IRB; Leica, Wetzlar, Germany).

### Reactive oxygen species (ROS)

Oxidative stress was achieved using hydrogen peroxide as previously described [26]. ROS levels were evaluated by DCFDA/H2DCFDA-Cellular ROS Assay Kit (Abcam, China) according to the manufacturer’s instructions. Cells were stained with Hoechst 33342 and imaged using a confocal laser scanning microscope with a × 40 oil objective (Leica DM IRB; Leica, Wetzlar, Germany). For the flow cytometric analysis of ROS levels, cells were analyzed using a flow cytometer (BD Biosciences, Franklin Lakes, USA).

### RNA isolation, reverse transcription, and real time PCR

Total RNA was isolated using TRIzol® reagent (15,596,018, Thermo Fisher Scientific, China). RNA concentration was measured by measuring the absorbance at 260 and 280 nm using NanoDrop 8000 spectrophotometer (NanoDrop technologies). cDNA was synthesized from 500 ng of total RNA sample using a RevertAid First Strand cDNA Synthesis Kit (K1622, Thermo Fisher Scientific, China). Real time PCR was performed using SYBR Green qPCR Master Mix (Life Technologies, China) on an ABI Prism 7500 Thermal Cycler (Applied Biosystems, Foster City, California, USA). The mRNA expression of the genes of interest was normalized against the housekeeping gene GAPDH. The difference between the mean Ct values of the gene of interest and the housekeeping gene was labelled ΔCt and the relative expression was calculated using the comparative Ct (2^−ΔΔCT^) method [27].

### hBMSC viability and live/dead assay

MTT cell proliferation assay and live/dead staining were used to assess cell viability as described above.

### Immunofluorescent staining and confocal microscopy

Immunofluorescent staining of Alkaline phosphatase (ALP) was used to assess osteoblastic maturation of hBMSCs after 14 days of culture in osteogenic medium. Briefly, hBMSCs were washed with PBS twice and fixed with 4% paraformaldehyde for 10 min at room temperature. Cells were permeabilized with 0.25% Triton X-100 for 10 min and blocked with 4% bovine serum albumin (BSA) for 1 h at room temperature. After rinsing with PBS twice, cells were incubated with rabbit polyclonal to ALP (1:100, ab224335, Abcam) overnight at 4 °C. The next day, cells were incubated with Fluorescein isothiocyanate-conjugated goat anti-rabbit IgG (H + L) secondary antibody for 1 h. Cells were counterstained with Alexa Fluor 594-labeled phalloidin followed by DAPI staining as described above. Images were acquired using a confocal laser scanning microscope with a × 40 objective (Leica DM IRB; Leica, Wetzlar, Germany).

### ALP and alizarin red S staining

ALP staining was also used to assess osteoblastic differentiation of hBMSCs after 14 days of culture in osteogenic medium. ALP staining was performed using BCIP/NBT Alkaline phosphatase Color Development Kit according to the manufacturer’s instruction (Beyotime, Shanghai, China). Images were taken using inverted light microscope with a × 10 objective (Leica, Wetzlar, Germany).

For the alizarin red staining, hBMSCs under osteoblastic differentiation were washed with PBS twice, then fixed by 4% paraformaldehyde for 20 min at room temperature. After fixation, the cells were then stained with 2% Alizarin Red S staining solution (pH = 4.1) for 30 min at room temperature. Images were taken using inverted light microscope with a × 10 objective (Leica, Wetzlar, Germany). Quantification of Alizarin Red S staining was performed according to previous study [28].

### Proteome profiler human XL cytokine array

Human XL cytokine array (Proteome Profiler Human XL cytokine arrays, ARY022, R&D Systems) was performed according to manufacturer’s instruction. hBMSCs were co-cultured with PLA or PLA-Exo scaffold for 3 days in osteogenic medium. Membranes were incubated with collected supernatant at 4 °C overnight. After a thorough washing, the membranes were incubated with a detection antibody cocktail for 1 h at room temperature and treated with streptavidin-horseradish peroxidase (HRP) solution for 30 min. The signal was visualized using enhanced chemiluminescence detection system and exposed to X-ray films. Images were captured and semi-quantified in the ImageJ software to determine the integrated density value of each protein spot on the grayscale.

### Statistical analysis

All data were expressed as mean ± standard deviations (SD, *n* = 3). Statistical analysis was performed using GraphPad Prism 7 (Version 7.02) for Windows (GraphPad Software Inc., USA). Statistical differences between groups were determined with one-way analysis of variance (ANOVA) with Bonferroni’s multiple comparison-tests. A value of *p* < 0.05 was considered statistically significant.

## Results and discussion

### Characterization of exosomes extracted from hBMSCs

**Fig. 1:**
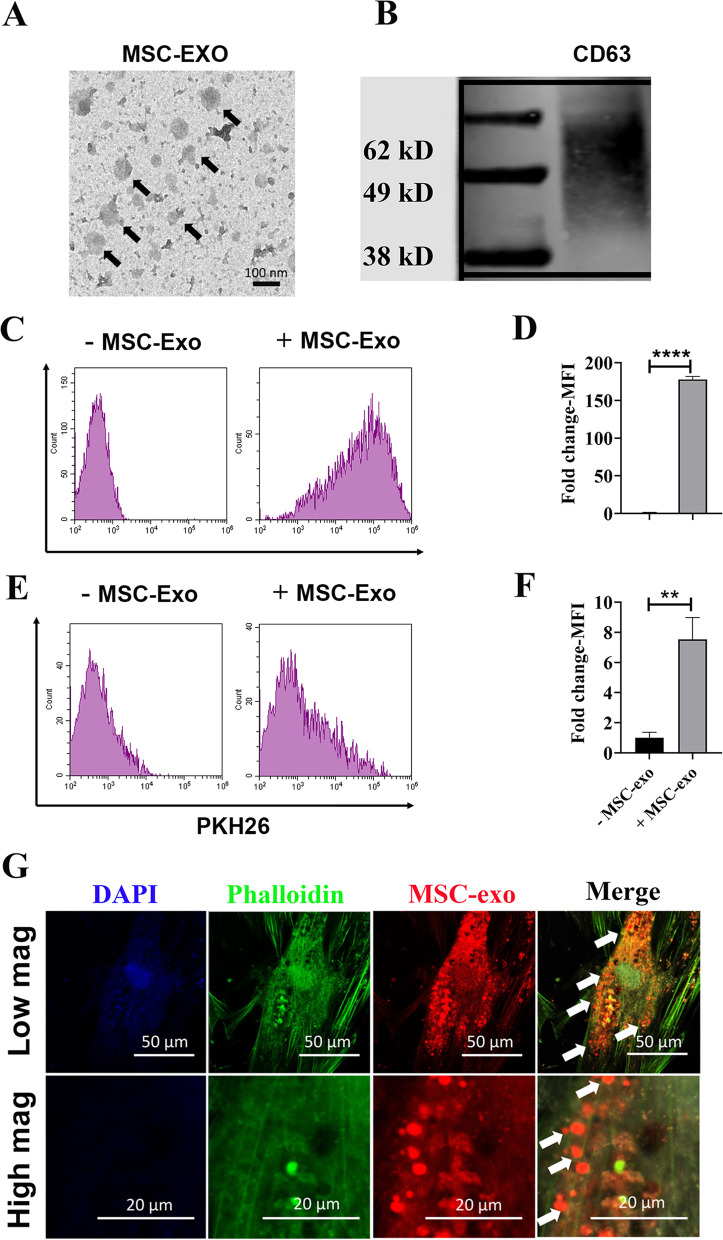
Characterization of hBMSCs-derived exosomes. **A** Morphological characterization of MSCs-derived exosomes with uranyl acetate negative staining. Isolated exosomes exhibited a cup-shaped morphology. **B** Western blot analysis of exosomal surface marker. Exosomes were found to be enriched for the exosomal surface marker CD63. **C** Flow cytometry analysis of exosome internalization into RAW264.7 cells. **D** The mean fluorescence intensity (MFI) obtained from RAW264.7 cells treated with or without MSC-Exo. **E** Internalization of MSC-Exo into hBSMCs. Cellular internalization of MSC-Exo by flow cytometric analysis. **F** MFI quantification of hBMSCs after 24 h of incubation with isolated exosomes. **G** Uptake of exosomes released from MSC-Exo by hBMSCs. Exosomes were prelabeled with PKH26. The internalization of the exosomes was evaluated by a confocal laser scanning microscopy

Despite the inherent tissue regenerative ability of hBMSCs, soluble factors secreted by MSCs, especially exosomes, have been indicated to play an important role in promoting tissue regeneration [29]. Due to their high therapeutic potential and their ability to transfer bioactive molecules, exosomes were firstly isolated from hBMSCs and were then used in this study to fabricate bio-inert PLA scaffold [29]. Exosomes were obtained from the conditioned medium of hBMSCs as previously described [22]. So far, various isolating methods have been developed to increase the yield of exosomes, including ultracentrifuge [30], commercial kits-based isolating method [31], ultrafiltration and precipitation [32], etc. Here, we used ultracentrifugation to isolate MSC-Exo from conditioned medium based on out established protocol [22]. To validate successful isolation of MSCs-derived exosomes, we used two different methods: TEM and western blot detecting exosomal marker. The isolated exosomes were first detected by TEM. As shown in Fig. 1A, TEM analysis of exosomes extracted from hBMSCs showed spherical or cup-shaped morphology, which is consistent with our previous result [22] and other people’s results [33, 34]. The western blotting result showed that isolated exosomes express significant levels of classical exosomal protein marker (CD63). (Fig. 1B).

To evaluate internalization of isolated exosomes into recipient cells, exosomes were labeled with PKH26 (red fluorescence) as described previously [35]. The mechanism of exosomes internalization into recipient cells remains a matter of debate, and several mechanisms have been proposed, including clathrin-dependent endocytosis, micropinocytosis, and caveolae-mediated endocytosis, etc. [36]. Raw 264.7 cells were incubated with PKH26-labeled exosomes for 24 h. The results from flow cytometry showed that macrophages treated with isolated exosomes demonstrated increased intracellular fluorescence signal, indicating that MSCs-Exo were uptake by RAW264.7 cells (Fig. 1C-D). Additionally, flow cytometry results also confirmed RAW264.7 cells have the higher uptake values compared to hBMSCs (Fig. 1E-F). It is also important to note that the uptake mechanism of exosomes from same origin is mainly recipient cells dependent, which exosomes released from donor cells are non-selectively incorporated into recipient cells [37]. We further used confocal microscopy to validate MSC-Exo internalization into recipient cells. As shown in Fig. 1G, PKH26 signal can be seen clearly around perinuclear areas in hBMSCs.

### Preparation of bioactive exosome-functionalized scaffolds

**Fig. 2:**
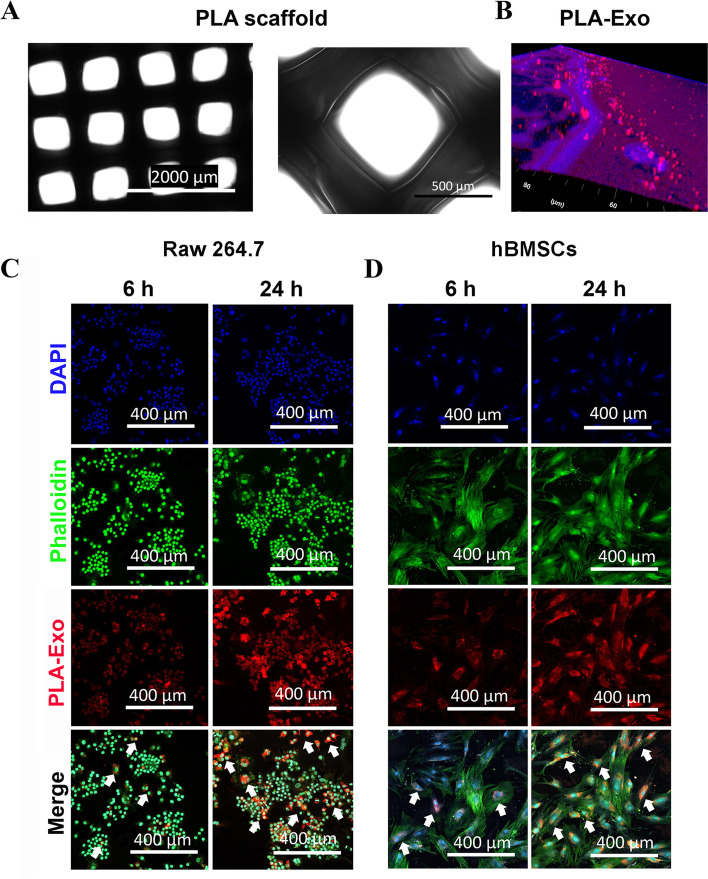
Fabrication of PLA-Exo scaffold. **A** The view of the 3D printed PLA scaffold. **B** Representative imaging of PLA scaffold modified with PKH26-labeled exosomes. **C** Fluorescent imaging of exosomes released from PLA-Exo scaffold into Raw 264.7 cells. Cells were stained with phalloidin (green), nucleus (blue), and examined by a confocal laser scanning microscopy. **D** Uptake of exosomes released from PLA-Exo scaffold by hBMSCs. Exosomes were prelabeled with PKH26. The internalization of the exosomes released from scaffold was evaluated by a confocal laser scanning microscopy

Cylindrical discs of PLA scaffolds with a nearly 55% porosity were firstly fabricated. Figure 2A shows a light microscopy image of a 3D printed PLA scaffold before MSC-Exo fabrication. The binding between MSC-Exo and 3D printed PLA scaffold were validated using a confocal laser scanning microscopy (Fig. 2B).

To examine the cellular internalization of released exosomes from PLA-Exo scaffold into recipient cells, PKH26-labeled exosomes were used for scaffold fabrication. It was noted that the red fluorescence signal increased dramatically at 24 h post-incubation in Raw 264.7 and hBMSCs, indicating good internalization of MSC-Exo from PLA-Exo scaffold into recipient cells (Fig. 2C&D).

### Macrophage viability and morphology

**Fig. 3:**
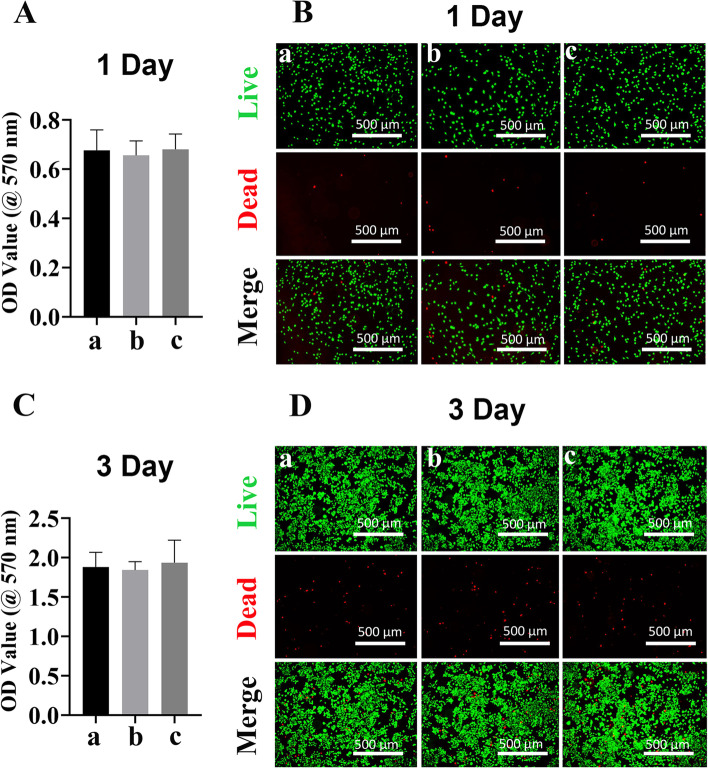
Effect of different scaffolds on Raw264.7 cells proliferation. **A-C** Cell proliferation via MTT assay in macrophages. Cell proliferation was evaluated by MTT assay at 1 day and 3 days. **B-D** Live and dead cells were assessed by a confocal laser scanning microscopy after labeling macrophages with fluorescein diacetate (FDA, green) and propidium iodide (PI, red). Green indicated living cells and dead cells were labeled as red fluorescence. Group a = control, group b = PLA scaffold, group c = PLA-Exo scaffold

To evaluate the viability of macrophages under the influence of different scaffolds, MTT cell proliferation assay was performed at different timepoints. It was reported that bone marrow aspirate MSCs-derived exosomes have a negative impact on the proliferation of activated peripheral blood mononuclear cells, and isolated B and T lymphocyte [38]. Abnormal proliferation of transforming growth factor (TGF)-β1-stimulated bronchial smooth muscle cell was also reduced following MSC-derived exosomes treatment [39]. Additionally, in a rat ischemia–reperfusion injury model, MSC-derived exosomes treatment was found to improve tubular epithelial cell proliferation [40]. Here, MTT assay results indicated that exposure of macrophages with PLA-Exo scaffold did not affect cell viability on day 1 (Fig. 3A). After 72 h of culture, MTT absorbance significantly increased among all groups, which showed no difference (Fig. 3C). These results indicated that no significant cytotoxicity of PLA/Exo scaffold on macrophages.

The viability and proliferation of macrophages under the influence of different scaffolds were then assessed using live/dead staining at different timepoints. Figure 3 B&D shows the fluorescence images of live and dead macrophages after co-culturing with different scaffolds for 1 day and 3 days. Macrophages cultured with PLA-Exo showed similar pattern and morphology as in groups without exosomes. When the cells were cultured for 3 days, the cell numbers significantly increased in all groups, while dead cells numbers showed similar pattern in the presence of exosomes as in groups without exosomes.

### Regulation of pro-inflammatory macrophages using PLA-Exo scaffolds

Macrophages are central phagocytic cells play an important role both in innate and adaptive immunity [41]. As important regulator to maintain normal tissue homeostasis, macrophages are located in almost all tissues with high plasticity and diversity [42]. Previous studies indicated resident osteal macrophages as well as migrated macrophages into the bone healing side have a major impact on the effective bone repair [43, 44]. Macrophages are generally divided into pro-inflammatory M1 and anti-inflammatory M2 types of based on their polarization states [45], which pro-inflammatory macrophages predominant the first stage of fracture healing while anti-inflammatory macrophages are abundantly detected in later stage of bone regeneration [46]. Excessive acute and chronic inflammation, caused by microbial infection, trauma, and autoimmune diseases, etc. lead to the persistent inflammatory state and excessive production of pro-inflammatory cytokines [47], which in turn, resulting in increased bone resorption and suppression of bone formation [47].

**Fig. 4:**
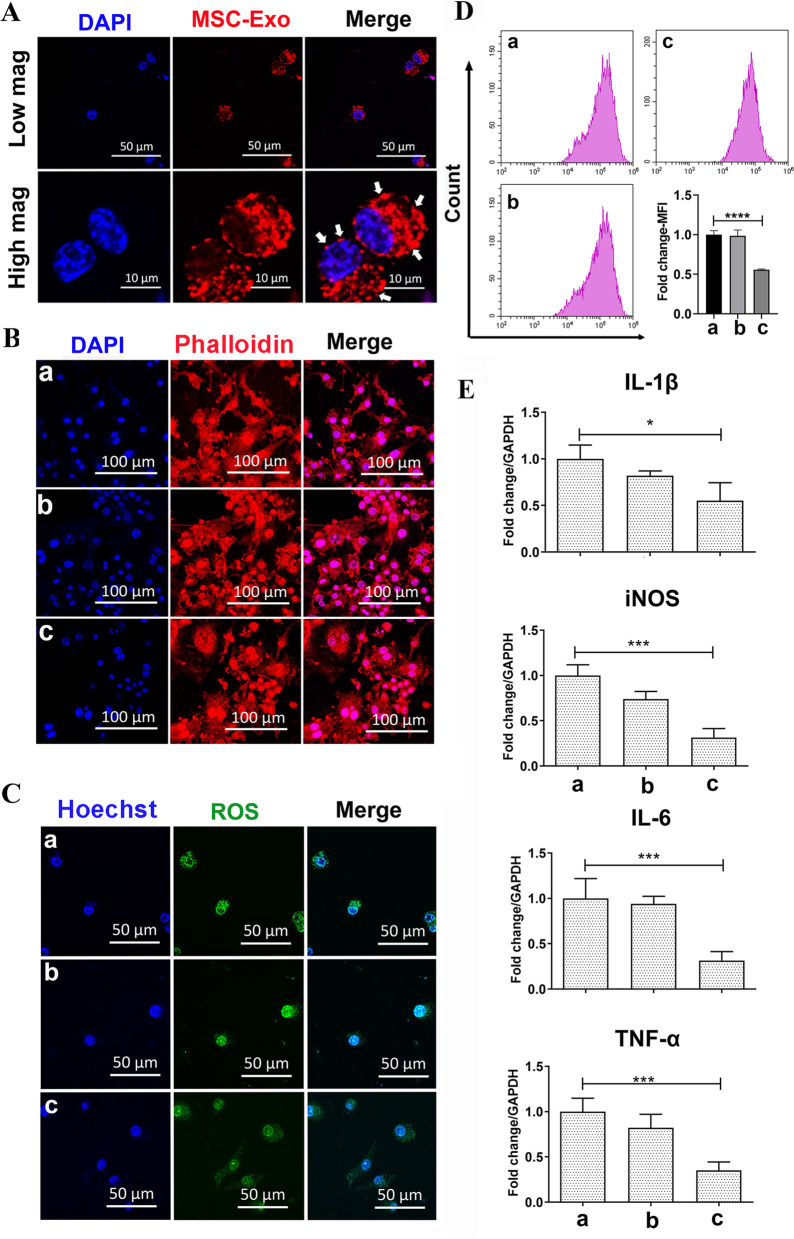
Effect of different scaffolds on the inflammatory macrophage polarization. **A** Confocal microscopy imaging of exosomes released from PLA-Exo scaffold internalization into pro-inflammatory macrophages. Cells were stained with DAPI (blue) and examined by a confocal laser scanning microscopy. **B** Representative confocal microscopy images of pro-inflammatory macrophages response to different scaffolds. Cells were fixed, stained for phalloidin (red), nuclei (blue) and visualized by a confocal laser scanning microscopy. **C** Representative CLSM images showing the changes of intracellular ROS levels. Nuclei were stained with Hoechst 33342 (blue). **D** ROS intensity detection using flow cytometry. Intracellular ROS levels decrease following PLA-Exo scaffold treatment: **** *p* < 0.0001. **E** qRT-PCR results of relative gene expression of pro-inflammatory cytokine. The values were normalized to GAPDH as a housekeeping gene. Significant difference (*p < 0.05, ****p < 0.0001). Group a = control, group b = PLA scaffold, group c = PLA-Exo scaffold

To examine the pro-inflammatory macrophage internalization of released exosomes, PKH carbocyanine dye-labeled exosomes were used for scaffold fabrication as mentioned above, and a confocal laser scanning microscopy was used for observation. As shown in Fig. 4A, PKH26 signal can be seen clearly around perinuclear areas in pro-inflammatory macrophages after 24 hours of culturing, which is consistent with previous study [48].

The macrophage morphology was then examined microscopically using a confocal laser scanning microscopy, focusing on the center regions of the samples. LPS predominantly polarizes macrophages to pancake-like shape after stimulation, with increased cellular areas and the number of processes. Previous studies indicated that pro-inflammatory M1 macrophages appeared large, round-shape morphology, while anti-inflammatory M2 macrophages showed elongated shape [49, 50]. Here, given the plasticity of macrophages, a hybrid morphology with both elongated and pancake-like shape can be seen (Fig. 4B).

Reactive oxygen species (ROS) and oxidative stress play an important role in a number of physiological and pathological conditions, including age-indued loss of bone mass and osteoporosis [51]. ROS act as one of the key molecules that regulate inflammatory signaling [52]. Therefore, we first examined the effects of PLA-Exo on ROS production in oxidative stress damage in macrophages by flow cytometry analysis. To examine ROS production, we used fluorescence staining following 2′,7′-dichlorodihydrofluorescein diacetate (DCFH-DA) staining. As shown in Fig. 4C, the intensities of green fluorescence in PLA-Exo scaffold group were significantly lower than those from control groups without exosome fabrication, indicating that the intracellular ROS level was reduced following PLA-Exo scaffold treatment. Xia et al. investigated the effect of MSCs-derived exosomes on H_2_O_2_-induced nucleus pulposus cell and found a significant reduction of ROS levels following exosome treatment [53]. In another study, MSCs-derived exosomes also decreased the levels iNOS expression and NO production IL-1β challenged chondrocytes [54]. To further validate our results, we used flow cytometry analysis. The results indicated that culture of oxidative stress-related macrophages with PLA-Exo scaffold significantly reduces relative ROS production (Fig. 4D).

We used qRT-PCR to determine the relative gene expression of pro-inflammatory cytokine after culturing with different scaffolds. Pro-inflammatory M1 macrophages are characterized by the high expression of inflammatory cytokines and [55]. As shown in Fig. 4E, IL-1β, iNOS, IL-6, and TNF-α gene expression levels were significantly decreased in PLA-Exo group compared with groups without exosome fabrication. Which is consistent with the anti-inflammatory effect reported in literature [56]. These results indicated that immunoregulatory role of PLA-Exo scaffold on pro-inflammatory response.

### hBMSC viability and morphology

**Fig. 5:**
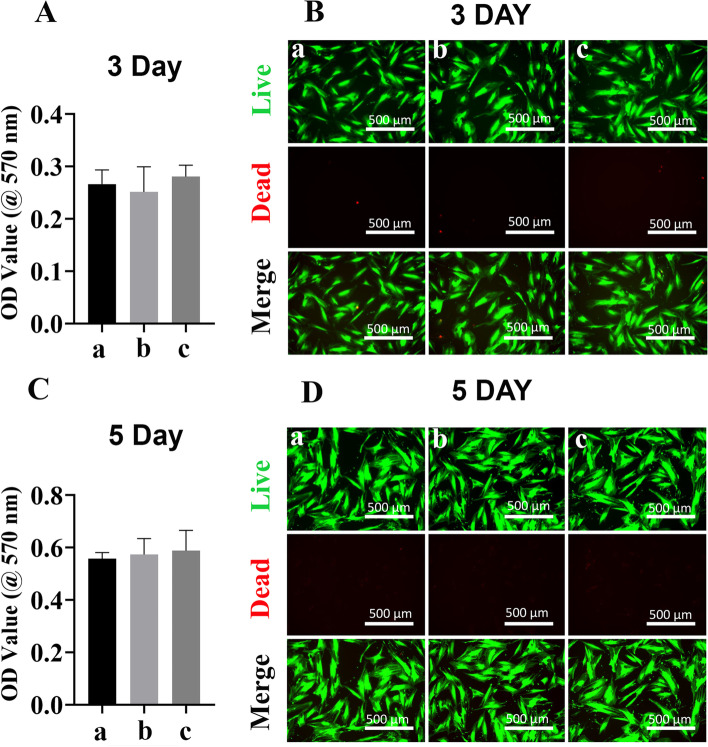
Cell proliferation analysis in hBMSCs. **A-C** Cell proliferation analysis. Cell viability was evaluated by MTT assay at 3 days and 5 days. **B-D** Live and dead assay of hBMSCs co-cultured with different scaffolds for 3 day and 5 days. The cells were stained with fluorescein diacetate (FDA, green) and propidium iodide (PI, red) and were examined by a confocal microscopy. Green indicated living cells and dead cells were labeled as red fluorescence. Group a = control, group b = PLA scaffold, group c = PLA-Exo scaffold

The biocompatibility of different scaffolds with hBMSCs was investigated using MTT cell proliferation assay at different timepoints. Previous study indicated that MSCs-derived exosomes could induce a dose-dependent increase of normal adult and diabetic wound fibroblasts proliferation and migration in vitro [57]. MSCs-derived exosomes have also been reported to enhance cell viability and proliferation in various cell-based models [58]. As shown in Figs. 5A&C, MTT assay results indicated that co-culture of hBMSCs with PLA-Exo scaffold showed no toxicity on day 3 and day 5. Sue l al. reported that both MSCs-derived exosomes and MSCs exosome immobilized PEI-modified electrospun fibers showed no stimulative effect on the proliferation of isolated T cells in vitro [59], which is consistent with our results. Our study also supported by the results from Qin et al., indicating marginal effect of MSCs-derived exosomes on osteoblast proliferation by flow cytometry analysis and MTT assay [60]. These results indicate that PLA-Exo scaffolds are highly biocompatible for hBMSCs growth.

We then performed live/dead staining to further assess cell proliferation after 3 day and 5 days culture. Figure 5B&D show hBMSCs had a homogeneous distribution and growth pattern in all groups. High viability of hBMSCs was also observed after culturing with PLA-Exo scaffold.

### Osteogenic differentiation of hBMSCs under the influence of PLA-Exo scaffolds

**Fig. 6:**
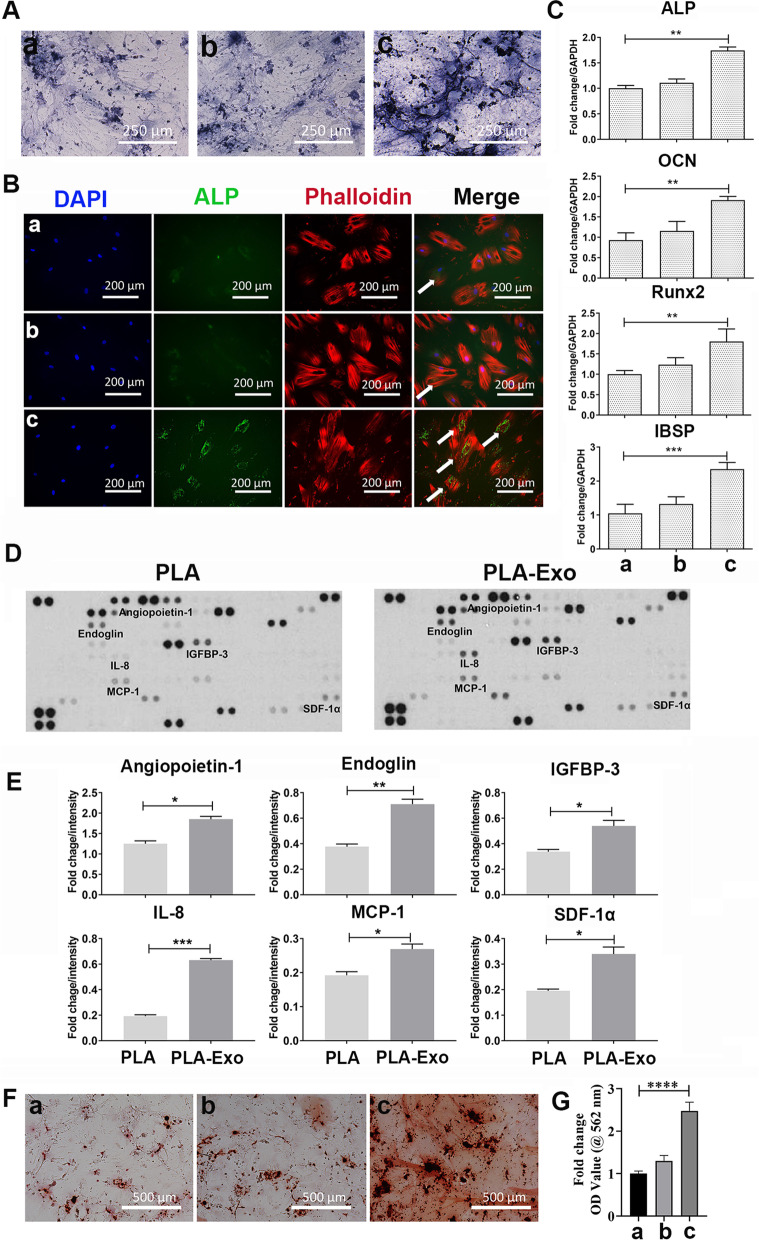
Osteogenic differentiation capacity of hBMSCs stimulated. **A** ALP staining images of hBMSCs cultured with different scaffolds at days 14. PLA-Exo scaffold improves the expression of ALP. **B** Immunofluorescence staining for osteogenic differentiation marker (ALP) in hBMSCs cultured with different scaffolds for 14 days. Cells were fixed, stained for ALP (green), phalloidin (red), and nuclei (blue), and examined by a confocal laser scanning microscopy. **C** Gene expression of osteogenesis-specific markers by qRT-PCR. Significant difference **p < 0.01, ***p < 0.001. **D** Representative images for cytokine array assay. hBMSCs were cultured with PLA or PLA-Exo scaffold for 3 days, and the cell culture supernatant were collected for human proteome XL cytokine array. **E** Quantification of cytokine array assay. Significant difference: *p < 0.05, **p < 0.01, ***p < 0.001). **F** Alizarin red S staining for calcium nodules formation at 21 days. **G** Calcium nodules formation was eluted and measured using a microplate reader at 405 nm. Significant difference: ****p < 0.0001. Group a = control, group b = PLA scaffold, group c = PLA-Exo scaffold

To evaluate the effect of PLA-Exo scaffold on the osteogenic differentiation of hBMSCs, we first evaluated the expression of ALP activity by using ALP staining. As an early expression marker associated with osteoblastic differentiation, ALP plays an important role in bone mineralization and skeletal development [61]. The effect of MSCs-derived exosomes on osteoblastic differentiation in vitro and in vivo were also reported [60, 62]. The ALP staining result is presented in Fig. 5A. The results indicated that PLA-Exo scaffold markedly increased ALP levels in hBMSCs compared to the control group after osteogenic induction for 14 days. To further evaluate the osteoblastic maturation of hBMSCs induced by the PLA-Exo scaffold, fluorescence staining was used to measure ALP expression, which were labeled with green fluorescence. As shown in Fig. 6B, the fluorescence intensity of ALP was significantly higher in hBMSCs treated with PLA-Exo scaffold compared with the rest groups.

The expression profile of osteogenic differentiation marker genes in hBMSCs were assessed using qRT-PCR. As shown in Fig. 6C, gene expressions of ALP, OCN, Runx2, and IBSP were examined, normalized by GAPDH. hBMSCs cultured with PLA-Exo scaffold expressed higher levels of transcription gene (Runx2). As one of the most important osteogenic transcription factors, Runx2 is crucial for the differentiation and maturation of MSCs [63]. hBMSCs treated with PLA-Exo scaffold also showed significant increase of the ALP mRNA expression, confirming the results of ALP staining and ALP fluorescence staining. In general, the gene expression levels of the other osteogenic differentiation markers (OCN and IBSP) also showed the same trend.

To understand the effect of PLA-Exo scaffold on the regulation of cytokines/chemokines secretion in hBMSCs, we performed cytokine array analysis. The expression of angiopoietin-1, endoglin, IGFBP-3, IL-8, MCP-1, and SDF-1α were significantly increased in PLA-Exo group (Fig. 6D&E). Suzuki et al. found that overexpression of angiopoietin-1 in osteoblasts enhanced bone mass in in angiopoietin-1-transgenic mice [64]. In addition, SDF-1α was reported involving in osteogenesis and angiogenesis, which its overexpression promoted bone regeneration in osteonecrotic femoral head [65].

To further validate the effect of PLA-Exo scaffold on the osteogenic differentiation of hBMSCs, matrix mineralization was assessed by Alizarin Red S staining. As shown in Fig. 6F, hBMSCs cultured with PLA-Exo scaffold exhibited more intense staining for calcium deposition. Consistently, quantification of the intensity of alizarin red S staining also indicated marked increase of calcium concentration in PLA-Exo scaffold (Fig. 6G).

## Conclusion

In the current study, a bioactive 3D porous PLA scaffold modified with MSC-Exo was fabricated to examine its roles in regulating inflammation and improving the osteogenic differentiation in hBMSCs. The bio-functionality of the original PLA scaffold was greatly improved by loading MSC-Exo, as evidenced by significant reduction of the pro-inflammatory markers and ROS production in inflammatory macrophages. Furthermore, the in vitro osteogenesis study further revealed high expression of osteoblastic markers and mineralization, indicating the pro-osteogenic effect of our bioactive 3D porous PLA scaffold. In the future, more studies, including the in vivo study, are necessary to further validate its bio-functionality.

## Data Availability

Please contact author for data requests.
